# Age-Related Changes of Adaptive and Neuropsychological Features in Persons with Down Syndrome

**DOI:** 10.1371/journal.pone.0113111

**Published:** 2014-11-24

**Authors:** Alessandro Ghezzo, Stefano Salvioli, Maria Caterina Solimando, Alice Palmieri, Chiara Chiostergi, Maria Scurti, Laura Lomartire, Federica Bedetti, Guido Cocchi, Daniela Follo, Emanuela Pipitone, Paolo Rovatti, Jessica Zamberletti, Tiziano Gomiero, Gastone Castellani, Claudio Franceschi

**Affiliations:** 1 Department of Experimental, Diagnostic and Specialty Medicine, University of Bologna, Bologna, Italy; 2 Associazione Nazionale Famiglie di Persone con Disabilitá Affettiva e/o Relazionale (ANFFAS) Onlus, Macerata, Italy; 3 Interdepartmental Centre “L. Galvani” – CIG, University of Bologna, Bologna, Italy; 4 Service for Disabled Students - University of Bologna, Bologna, Italy; 5 Fondazione Don Carlo Gnocchi-Onlus, Falconara (An), Italy; 6 Operative Unit of Neonatology – Department of Medical and Surgical Sciences, University of Bologna, Bologna, Italy; 7 CEPS Centro Emiliano studi sociali per la trisomia 21, Bologna, Italy; 8 Azienda USL Bologna, Bologna, Italy; 9 Comune di Bologna - Settore Salute, Sport e Città Sana, Bologna, Italy; 10 Project DAD (Down Alzheimer Dementia), Associazione Nazionale Famiglie di Persone con Disabilitá Affettiva e/o Relazionale (ANFFAS) Trentino Onlus, Trient, Italy; 11 Department of Physics, University of Bologna, Bologna, Italy; 12 IRCCS Institute of Neurological Sciences, Bologna, Italy; 13 National Research Council of Italy, CNR, Institute for Organic Synthesis and Photoreactivity (ISOF), Bologna, Italy; 14 National Research Council of Italy, CNR, Institute of Molecular Genetics, Unit of Bologna, Bologna, Italy; Tokai University, Japan

## Abstract

Down Syndrome (DS) is characterised by premature aging and an accelerated decline of cognitive functions in the vast majority of cases. As the life expectancy of DS persons is rapidly increasing, this decline is becoming a dramatic health problem. The aim of this study was to thoroughly evaluate a group of 67 non-demented persons with DS of different ages (11 to 66 years), from a neuropsychological, neuropsychiatric and psychomotor point of view in order to evaluate in a cross-sectional study the age-related adaptive and neuropsychological features, and to possibly identify early signs predictive of cognitive decline. The main finding of this study is that both neuropsychological functions and adaptive skills are lower in adult DS persons over 40 years old, compared to younger ones. In particular, language and short memory skills, frontal lobe functions, visuo-spatial abilities and adaptive behaviour appear to be the more affected domains. A growing deficit in verbal comprehension, along with social isolation, loss of interest and greater fatigue in daily tasks, are the main features found in older, non demented DS persons evaluated in our study. It is proposed that these signs can be alarm bells for incipient dementia, and that neuro-cognitive rehabilitation and psycho-pharmacological interventions must start as soon as the fourth decade (or even earlier) in DS persons, *i.e.* at an age where interventions can have the greatest efficacy.

## Introduction

Down Syndrome (DS) is the most common known genetic cause of moderate to severe intellectual disability, with a prevalence of around 14 per 10,000 live births [1], [2]. The life expectancy for DS subjects has dramatically changed in the last decades, increasing from 12 in 1949 to nearly 60 years of age today [3], [4], and it is believed that it will increase in the future [5]. This implies the occurrence of an unprecedented phenomenon of aging in the DS population, which in Italy is estimated to be about 49,000 persons [6]. DS is characterised by premature aging, especially in the immune system and the CNS, DS persons experience age-related changes in cerebral functions much earlier than non-trisomic people. Most of the DS subjects begin to develop Alzheimer-like neuro-pathological signs, as early as 30 years of age, such as plaque and tangle formation in the amygdala, hippocampus, and association areas of the frontal, temporal, and parietal cortex [7], which are claimed to occur in all people with DS by 40 years of age [8]–[11]. Dementia is present in 55% of DS adults in their sixth decade [12], preceded by changes in language skills [13], [14] and in frontal lobe functions [15] such as executive functions [13].

In this study we have thoroughly investigated the neuropsychological, cognitive and neuropsychiatric aspects in a group of 67 persons with DS of different ages, from 11 to 66 years, with the aim of characterising the accelerated age-associated decline of cognitive performances of these persons. The main result of this investigation is that the neuropsychological functions and adaptive skills are lower in older DS persons and this impairment precedes the overt stage of dementia. In order to counteract the neurodegenerative process, the evaluation of such parameters may serve as the basis for both pharmacological and rehabilitative interventions, performed in a specific and individually tailored way.

## Materials and Methods

### Participants

Individuals with DS were enrolled prospectively from 2008, in an open-label study on cognitive decline in DS. The study was approved by the local Ethical Committee (S. Orsola Hospital, University of Bologna; ethical clearance #126/2007/U/Tess, released on December 18, 2007). Written informed consent to participate in the study was obtained from adult DS persons and from parents or authorised tutors for those under age. Written informed consent was also obtained for adult DS persons from parents or relatives (brothers/sisters). Subjects were recruited with the help of CEPS, OPIMM and ANFFAS, three local non-profit associations dealing with DS persons operating in the eastern part of Emilia-Romagna Region (Bologna and Ferrara provinces). Participation in the study was on a totally voluntary basis, with no reward for the participants or their families. Exclusion criteria were current acute illnesses, hepatic, renal or cardiac insufficiency, consumption of antioxidant or nutraceutical substances (vitamins, lipoic acid, acetylcysteine, omega 3 and 6 fatty acids, probiotics) within the last two months. A total of 81 families with a DS person were approached for this study. Four families refused to participate in the study after the first meeting of introduction and explanation of the study or after the first clinical interview. A total of 77 families agreed to participate in the study and the DS persons underwent blood tests and neuropsychiatric evaluation. Ten DS persons were excluded from further neuropsychological evaluation because of the presence of features of dementia, or other severe psychiatric features according to DSM IV criteria [16] or other adverse clinical conditions that could interfere with the evaluation.

The relatives or caregivers of the DS persons were asked to present a diagnosis of DS based on karyotype. This was obtained in 64 out of 67 persons. Of these, 50 were trisomy 21, 11 were mosaics, and 3 were translocations. In 44 out of 67 persons it was possible to perform a complete clinical, neuropsychological, and cognitive assessment, while the remaining 23, because of cognitive impairment and/or low level of collaboration, were able to complete appropriately only a part of the cognitive and neuropsychological tests.

### Assessment protocol

Each DS person underwent an objective observation by two physicians (F.B and G.C.) assessing anthropometric parameters such as height, weight and Body Mass Index (BMI) and others. During the visit, a peripheral blood donation (of about 30 ml) was taken to assess ApoE genotype and the main blood biochemical parameters. General clinical data were collected by a neuropsychiatrist (AG) with experience in the field of neurocognitive impairment, by using a semistructural interview with parents or caregivers regarding family medical history, personal medical history, socio-economic data collection, education level and social activities.

### Cognitive, neuropsychological, and adaptive behaviour assessment

The evaluation of neuropsychological functions, cognitive level, language skills and adaptive behaviour, in relation to the different age groups, was conducted in two separate sessions by two psychologists trained in the field of mental retardation (MCS and AP) using the following test battery: WISC-III [17], WAIS-R [18], Spatial Span [19], Categorical fluency [20], Token Test [21], Phonological fluency [22], Tower of London [23]. Frontal Assessment Battery (FAB) [24] Italian version [25], The Visual Object and Space Perception Battery – VOSP [26], Vineland Adaptive Behaviour Scales (VABS) [27]. DSQIID Questionnaire (Dementia Screening Questionnaire for Individuals with Intellectual Disabilities, [28]–[30] was used by a neuropsychiatrist (AG) to detect dementia features (see Supplemental materials and methods in File S1 for further details). Laterality assessment was performed by an experienced psychomotricist (CC).

### ApoE genotyping

In order to exclude that possible cognitive decline was associated with known genetic risk factors, all the subjects were genotyped for ApoE, as the ε4 allele is a known risk factor for Alzheimer's Disease (AD) both in general population [31] and in DS people [32]. ApoE genotyping was performed on persons with DS, siblings and mothers as previously described [33]. Briefly, specific primers, F4 (5′-ACAGAATTCGCCCCGGCCTGGTACACAC-3′) and F6 (5′-TAAGCTTGGCACGGCTGTCCAAGGA-3′) described by Emi et al. [34], were used to amplify by PCR the region containing the Arg/Cys polymorphism at codons 112 and 158 of the APOE gene. Standard PCR reaction was carried out in 30 µl volume containing 200 ng of genomic DNA, 0.3 µM of each primers, 0.25 mM of dNTPs mix, 1X of buffer 10X, 1.5 mM of MgCl2, 10% DMSO 100% with 1.5 U Taq polymerase. Cycling was performed for 45 cycles of denaturation at 95°C for 1 min, annealing at 63°C for 1 min and extension at 70°C for 2 min. An initial denaturing step at 95°C for 5 min and a final extension step at 70°C for 12 min were also carried out. Amplified products were digested overnight at 37°C with 3.5 U of the restriction enzyme HhaI and visualized via ethidium bromide staining of 4.5% agarose Molecular Screening gels. Each of the isoforms was distinguished by a unique combination of HhaI fragment sizes that enabled unambiguous typing of all homozygotic and heterozygotic combinations. HhaI cleaves at GCGC encoding 112Arg (E4) and 158Arg (E3, E4), but does not cut at GTGC encoding 112Cys (E2, E3) and 158Cys (E2).

### Statistical analysis

The analysis includes 67 patients, 34 males and 33 females, with an age range from 11 to 66 years. Numeric variables have been tested for normal distribution. Two kinds of analysis were carried out:

For the whole group, variable “age” has been compared to the neuropsychological and adaptive parameters by using the Pearson parametric correlation for normally distributed continuous variables or the Spearman non-parametric correlation in all other cases.

For the 59 patients over 18 years, a comparison between the 3 age groups (N = 24 aged 18–29 years, N = 17 aged 30–39 years, N = 18 aged over 40 years) was performed using the ANOVA or the equivalent non parametric test (Wilcoxon Mann Whitney). For normally distributed variables we used ANOVA test and the differences across the groups means were tested with Tukey-Kramer multiple comparisons test, because the group sizes are different. For the non-normally distributed variables we used the ANOVA non-parametric test (Kruskal-Wallis test) and the differences across group means were tested with Bonferroni multiple comparisons test.

Categorical variables (DISQIID categories, laterality) were tested with the chi-square test or the Fisher exact test if the expected value for each cell was lower than five.

Because of the high number of psychological tests considered in the analysis, the Benjamini and Hochberg False Discovery Rate (FDR) test [35] were also performed for every group of variables. A FDR <0.05 was considered to be statistically significant. Statistical analysis was performed using SAS v. 9.2.

## Results

As mentioned above, we evaluated only non-demented persons with DS, with no severe clinical conditions that could affect the neuropsychological assessment. All the raw data can be found in the Database File S2. Demographic and anthropometric measures for the 67 persons with DS are reported in Table 1, where data on the whole group as well as 4 separate age groups are given. This division was decided on the basis of the fact that after 30 years of age DS persons are expected to develop Alzheimer-like neuro-pathological signs, as mentioned above [7]; and virtually all those over 40 are claimed to display these signs [8]–[11]. Younger DS persons were subdivided in two groups as those under 18 years of age are expected to attend school.

**Table 1:** Demographic and clinical features of the studied DS persons.

DEMOGRAPHICS	The whole group (n = 67)	11–17 years (n = 8)	18–29 years (n = 24)	30–39 years (n = 17)	≥40 years (n = 18)
**Age (years)**	31.62±13.25	13.96±2.21	22.34±3.40	34.27±3.04	49.34±6.91
**Range**	11–66	11–17	18–29	30–39	40–66
**Gender (M, F)**	M = 34 (50.7%)	M = 2 (25%)	M = 13 (54.2%)	M = 11 (64.7%)	M = 8 (45.4%)
	F = 33 (49.3%)	F = 6 (75%)	F = 11 (45.8%)	F = 6 (35.3%)	F = 10 (55.6%)
**Weight (Kg)**	60.24±13.77	48.57±10.94	59.88±13.16	60.47±15.40	65.01±11.95
**Height (cm)**	149.83±9.06	144.71±13.35	149.48±7.79	154.30±7.63	148.27±8.84
**BMI**	26.87±5.41	23.20±5.00	26.56±4.17	25.64±5.62	29.76±5.72
**Abdominal circumferences (cm)**	88.53± 14.21	76.13±7.51	84.79±13.86	89.50±15.02	98.16±9.94
**Frontal-occipital head circumference (cm)**	52.20±1.75	51.10±1.41	52.54±1.51	52.69±1.97	51.69±1.80
**Cognitive impairment levels**					
Mild	34 (51%)	3 (37.5%)	14 (58.5%)	10 (59%)	7 (39%)
Moderate	17 (25%)	2 (25%)	7 (29%)	4 (23.5%)	4 (22%)
Severe	9 (13.5%)	3 (37.5%)	1 (4%)	2 (11.5%)	3 (17%)
Profound	7 (10.5%)	0	2 (8.5%)	1 (6%)	4 (22%)
ApoE genotyping	n = 66	n = 8	n = 23	n = 17	n = 18
ε2/ε3	6 (9%)	0	1 (4.5%)	4 (23%)	1 (5.5%)
ε2/ε4	1 (2%)	0	1 (4.5%)	0	0
ε3/ε3	49 (74%)	6 (75%)	15 (65%)	12 (71%)	16 (89%)
ε3/ε4	10 (15%)	2 (25%)	6 (26%)	1 (6%)	1 (5.5%)
ε4/ε4	0	0	0	0	0
**Visual problem**					
No problem	13 (19.4%)	0	1 (4.2%)	4 (23.5%)	8 (44.4%)
Mild	45 (67.2%)	8 (100%)	19 (79.2%)	9 (53%)	9 (50%)
Moderate	9 (13.4%)	0	4 (16.6%)	4 (23.5%)	1 (5.6%)
Severe	0	0	0	0	0
**Hearing problem**					
No problem	61 (91%)	8 (100%)	22 (91.6%)	14 (82.3%)	17 (94.4)
Mild	4 (6%)	0	1 (4.2%)	2 (11.8%)	1 (5.6)
Moderate	2 (3%)	0	1 (4.2%)	1 (5.9%)	0
Severe	0	0	0	0	0
**Congenital Heart Disease**	17 (25.4%)	3 (37.5%)	8 (33.3%)	6 (35.3%)	0
**Tyroxine treatment**	31 (46.3%)	3 (37.5%)	10 (41.7%)	8 (47%)	10 (55.6%)
**Antidepressant Treatment**	2 (3%)	0	0	2 (11.7%)	0
**Anti-psychotic**	6 (9%)	0	0	2 (11.7%)	4 (22.2%)
**Epilepsy**	2 (3%)	0	1 (4.2%)	0	1 (5.6%)
**Laterality**	A	B	C	D	E
**Arm**	Right 44 (69.8%)	Right 5 (62.5%)	Right 13 (56.5%)	Right 14 (87.5%)	Right 12 (75%)
(A = 63; B = 8; C = 23; D = 16; E = 16)	Left 12 (19%)	Left 1 (12.5%)	Left 8 (34.8%)	Left 1 (6.25%)	Left 2 (12.5%)
	Mixed 7 (11.1%)	Mixed 2 (25%)	Mixed2 (8.7%)	Mixed 1 (6.25%)	Mixed 2 (12.5%)
**Leg**	Right 19 (31.7%)	Right 3 (37.5%)	Right 7 (31.8%)	Right 7 (43.7%)	Right 2 (14.3%)
(A = 60, B = 8; C = 22; D = 16; E = 14)	Left 18 (30%)	Left (25%)	Left 10 (45.5%)	Left 3 (18.8%)	Left 3 (21.4%)
	Mixed 23(38.3%)	Mixed 3 (37.5%)	Mixed 5 (22.7%)	Mixed 6 (37.5%)	Mixed 9 (64,2%)
**Eye**	Right 40 (66%)	Right 5 (62.5%)	Right 16 (72.7%)	Right 9 (56.2%)	Right 10 (66.6%)
(A = 61, B = 8; C = 22; D = 16; E = 15)	Left 21 (34%)	Left 3 (37.5%)	Left 6 (27.3%)	Left 7 (43.8%)	Left 5 (33.4%)
**Education (years)**	9.63±3.05	8.13±1.89	12.25±1.51	9.41±2.45	7±2.79
**Current Accommodation**					
On his/her own	1 (1.5%)	0	0	1 (6%)	0
With relatives	58 (86.5%)	8 (100%)	24 (100%)	15 (88%)	11 (61.1%)
In a group home with full time staff	6 (9%)	0	0	0	6 (33.3)
In a nursing home with full nursing care	2 (3%)	0	0	1 (6%)	1 (5.6%)
**Daily activity/Work**					
School	11 (16.4%)	8 (100%)	3 (12.5%)	0	0
Mainstream Paid Employment	17 (25.4%)	0	10 (41.7%)	6 (35.3%)	1 (5.6%)
Sheltered workshop (work centre)	23 (34.3%)	0	10 (41.7%)	5 (29.4%)	8 (44.4%)
Day centre for people with learning disabilities	13 (19.4%)	0	1 (4.1%)	6 (35.3%)	6 (33.3%)
No specific activity	3 (4.5%)	0	0	0	3 (16.7%)

Values are expressed as mean ± SD. See text for further description.

Our data are in line with previous research reporting that about 50% of both male and female DS adults are overweight [36]. On the basis of the clinical interview, most of the persons were reported to have mild to moderate visual problems; in our sample no hearing problems were reported in the vast majority of subjects. About half of the persons were on thyroid hormone replacement therapy and one fifth of the persons over 30 years of age were in antipsychotic and/or antidepressant therapy. The vast majority of the persons included in this study live with relatives or in a group home with full time staff. Most of the persons have an active life, in mainstream paid employment or in work centres (especially people from 18 to 39 years of age).

### ApoE genotype analysis

On the basis of this analysis the group of DS persons was not found to have an unevenly high frequency of the APOE4 allele, a recognised risk factor for AD. Results are reported in Table 1. The majority of the subjects carried a ε3/ε3 genotype, in agreement with the frequencies found in the Italian population. No carrier of the risk genotype ε4/ε4 was found in the group of DS persons. The frequency of ε4 allele was quite high (15%) with respect to that usually found in the Italian population (about 7%), and it is similar to that found by Prasher et al. in an English DS cohort (13,7%) [37]. However, no conclusion can be drawn, due to the small size of the sample.

### Cognitive levels

Four levels of Cognitive Impairment (mild, moderate, severe and profound) according DSM IV criteria, were assessed (Table 1). Forty-four DS subjects were tested for the cognitive level with Wechsler Scale and found to be within the range of Cognitive Impairment (IQ scores below 70), as expected (Table 2). For the remaining 23 persons with DS, the cognitive level was assessed by using the mental age values extrapolated from VABS. The gross results of the evaluation of the neuropsychological and adaptive functions are summarized in Table 2, where data are presented for the whole group as well as for groups of different age. Further tables focus in more detail on specific parameters.

**Table 2:** neuropsychological, adaptive and cognitive evaluation of the DS persons.

	The whole group = A	11–17 years = B	18–29 years = C	30–39 years = D	≥40 years = E
**SHORT MEMORY SPAN**					
**Spatial span (Corsi Test)**	2.27±1.43	2.75±1.39	2.75±1.39	2.14±1.46	1.47±1.18
**[A = 63; B = 8; C = 24; D = 14; E = 17]**					
**Digit span forward**	2.42±1.20	3.25±0.46	2.46±1.22	2.40±1.30	2.00±1.19
**[A = 65; B = 8; C = 24; D = 15; E = 18]**					
**LANGUAGE**					
**Semantic fluency**	25.18±13.19	30.75±8.51	29.79±13.21	25.50±12.74	15.76±10.91
**[A = 65; B = 8; C = 24; D = 16; E = 17]**					
**Comprehension (Token test)**	18.27±8.70	21.00±5.15	21.35±8.52	19.50±9.05	11.92±6.87
**[A = 65; B = 8; C = 24; D = 15; E = 18]**					
**EXECUTIVE FUNCTIONS**					
**Phonemic fluency**	5.74±5.46	7.62±3.29	7.79±6.65	6.19±4.58	1.53±2.00
**[A = 65; B = 8; C = 24; D = 16; E = 17]**					
**Digit span backward**	1.05±1.24	1.37±1.60	1.17±1.37	1.47±0.92	0.39±0.92
**[A = 65; B = 8; C = 24; D = 15; E = 18]**					
**Tower of London (n°perfect answers)**	3.13±2.99	3.57±2.51	4.23±2.65	3.31±3.59	1.35±2.29
**[A = 62; B = 7; C = 22. D = 16. E = 17]**					
**FRONTAL ASSESSMENT BATTERY-FAB**	n = 59	n = 8	n = 19	n = 15	n = 17
** FAB total score**	7.64±3.69	10.25±2.25	8.37±3.71	8.80±3.43	4.59±2.50
**Similarity**	1.03±0.91	1.62±0.52	1.10±1.00	1.07±0.88	0.65±0.86
**Phonological fluency**	0.49±0.65	0.87±0.64	0.68±0.75	0.53±0.64	0.06±0.24
**Motor series**	1.86±1.14	2.75±0.71	1.74±0.93	2.27±1.03	1.23±1.25
**Conflictual instructions**	0.85±1.00	1.38±1.06	1.05±1.08	0.87±1.06	0.35±0.61
**Go-no go**	0.83±1.02	1.13±1.36	1.10±1.00	1.07±1.10	0.18±0.39
**Prehension behaviour**	2.63±0.89	2.88±0.35	2.68±0.75	3.00±0.00	2.12±1.32
**VISUAL OBJECTS and SPACE PERCEPTIONS BATTERY-VOSP (cut-off)**	n = 47	n = 4	n = 20	n = 15	n = 8
**Incomplete Letters (17)**	16.11±2.88	17.50±1.00	16.40±2.44	16.40±2.26	14.13±4.64
**Silhouettes (16)**	11.94±4.76	15.25±3.86	13.80±4.53	9.07±3.88	11.00±4.66
**Object Decision (15)**	12.47±3.13	14.75±2.50	12.30±3.25	12.60±2.75	11.50±3.74
**Progressive Silhouettes (14)**	9.02±3.46	10.00±2.58	9.25±3.64	8.33±4.05	9.25±2.31
**Dot Counting (8)**	7.15±2.93	7.75±0.96	7.70±2.58	7.67±2.69	4.50±3.74
**Position Discrimination (18)**	13.64±4.33	13.50±5.00	14.05±3.20	13.53±4.52	12.88±6.51
**Number Location (7)**	2.06±2.45	4.00±1.41	2.70±2.77	1.00±1.36	1.50±2.83
**Cube Analysis (6)**	4.47±2.22	5.00±1.83	5.00±2.18	4.67±2.19	2.50±1.77
**Total score**	76.85±17.13	87.75±11.76	81.20±15.87	73.27±15.52	67.25±21.19
**VINELAND ADAPTIVE BEHAVIOUR SCALE**	n = 67	n = 8	n = 24	n = 17	n = 18
**COMMUNICATION**	106.48±15.02	108.00±9.26	109.50±14.18	107.71±16.10	100.61±16.51
**Receptive**	105.67±14.49	109.88±8.37	110.50±10.90	106.94±12.89	96.17±18.19
**Expressive**	104.82±14.22	105.25±10.61	107.04±14.23	104.71±14.90	101.78±15.47
**Written**	107.72±15.33	110.00±11.21	111.25±14.90	110.41±15.32	99.44±15.49
**DAILY LIVING SKILLS**	114.34 ±14.60	112.38±8.37	118.29±10.23	117.71±17.11	106.78±16.91
**Personal**	108.40±11.85	112.00±5.37	110.96±8.57	110.47±11.90	101.44±15.15
**Domestic**	115.61±14.86	114.63±10.90	121.46±10.51	116.35±17.94	107.56±15.45
**Community**	113.36±18.96	108.88±11.23	116.63±18.10	120.53±20.87	104.22±18.03
**SOCIALIZATION**	114.91±17.24	118.13±9.69	119.04±13.97	119.88±17.90	103.28±18.79
**Interpersonal relationship**	113.03±15.64	117.50±14.07	116.46±15.79	116.47±11.90	103.22±16.05
**Play and Leisure time**	109.82±18.11	115.88±8.36	115.00±14.12	114.12±18.30	96.17±19.70
**Coping Skills**	116.52±14.95	118.13±9.72	118.83±12.52	119.18±16.57	110.22±17.35
**MOTOR SKILLS**	108.60±9.37	110.25±4.68	110.67±6.59	109.00±11.65	104.72±11.03
**Gross Motor Skills**	107.45±9.09	109.75±4.30	109.83±6.91	107.59±11.68	103.11±9.46
**Fine Motor Skills**	108.64±10.63	109.25±7.72	110.75±7.93	108.76±12.14	105.44±13.15
**Vineland Total Score (SQ)**	113.18±13.90	114.38±7.76	116.75±10.81	116.00±15.01	105.22±16.15
**WECHSLER SCALE**	n = 44	n = 8	n = 21	n = 10	n = 5
**VERBAL IQ**	49.82±14.34	48.25±10.08	53.43±13.02	51.60±12.91	33.60±20.02
**PERFORMANCES IQ**	48.82±14.14	44.88±9.28	51.38±12.49	52.90±12.44	36.20±23.72
**TOTAL IQ**	46.23±13.82	42.00±11.25	49.71±12.69	48.80±11.84	33.20±19.60

The values are expressed as means ± SD. In square brackets the number of subjects evaluated for the whole group and subdivided for age. The whole group  = A; 11–17 years group  = B; 18–29 years group  = C; 30–39 years group  = D; ≥40 years group = E.

### Neuropsychological profile

**Figure 1:**
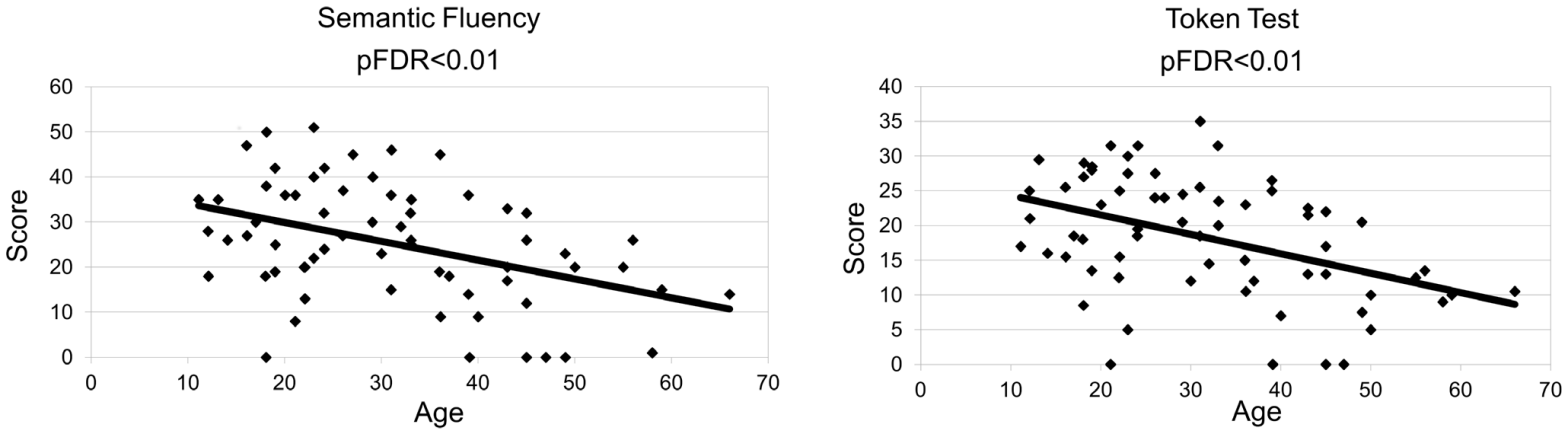
Correlations between DS language test (Semantic Fluency, Token Test) scores and age (expressed in years). All the neuropsychological values are inversely correlated with the age. pFDR<0.05 significant, pFDR<0.01 highly significant. More details are reported in Table 3.

**Figure 2:**
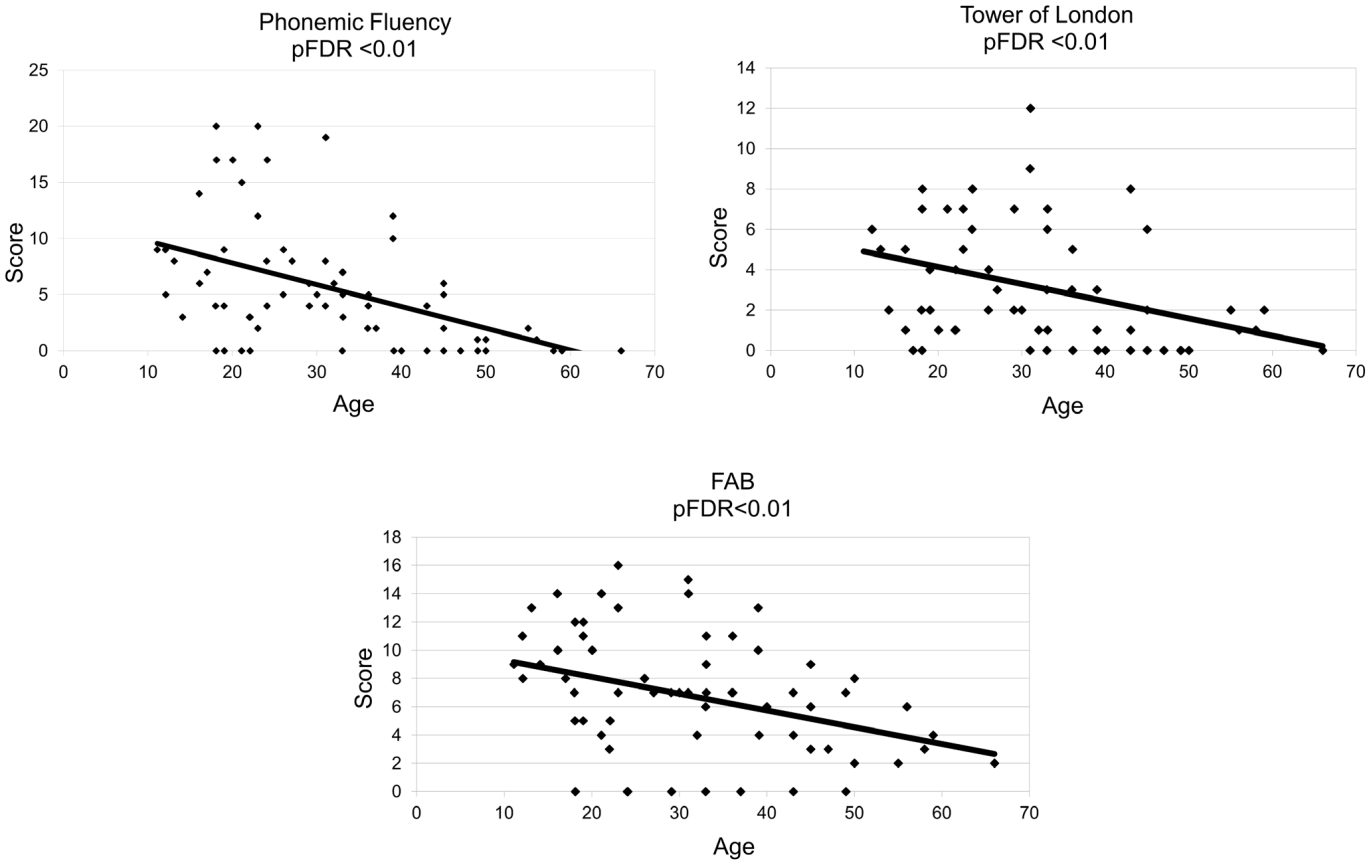
Correlations between DS Executive Functions test scores (Phonemic Fluency, Tower of London, FAB total scores) and age (expressed in years). All the values are inversely correlated with the Age. pFDR<0.05 significant, pFDR<0.01 highly significant. More details are reported in Table 3 and Table 4.

A number of tests for the neuropsychological evaluation of the DS persons were performed. For each of them the number of persons who scored 0 (null) is reported in Table S1 in File S1. When performing short memory and language test, we observed a significant inverse correlation between age and semantic fluency, and token test (Table 3, Figure 1). Spatial span and digit span forward tests were also found to be inversely correlated with age (Table 3). With regard to executive functions, Phonemic fluency and Tower of London also inversely correlated with age (Table 3, Figure 2).

**Table 3:** Analysis of Short Memory Span, Language and Executive functions.

	Spatial span (Corsi)*	Digit span forward*	Semantic fluency**	Token test*	Phonemic fluency*	Digit span backward*	Tower of London*
Coeff	−0.37684	−0.31992	−0.41995	−0.42148	−0.47846	−0.26861	−0.39824
P	**0.0023**	**0.0094**	**0.0005**	**0.0005**	**<0.0001**	**0.0305**	**0.0013**
FDR	**0.0107**	**0.0282**	**0.0042**	**0.0042**	**0.0014**	0.0645	**0.0078**
N	63	65	65	65	65	65	62

Coefficient of correlation with age (coeff), False Discovery Rate value (FDR) and number of evaluated DS persons are reported for each item. In bold significant p values are reported. * =  Spearman non-parametric correlation. ** =  Pearson parametric correlation.

The executive functions were further evaluated by using FAB, and also in this case we observed a significant inverse correlation with age in the total FAB score (Table 4, Figure 2). We also observed that the score of the phonological fluency subtest was not only very low when compared to other FAB items (Table 2), but also showed the highest correlation with age (Table 4, p<0.01).

**Table 4:** Analysis of Frontal Assessment Battery (FAB).

	FAB Total score**	Similarity*	Phonological fluency*	Motor series*	Conflictual instructions*	Go-no go*	Prehension*
Coeff	−0.51787	−0.31709	−0.49816	−0.30420	−0.29750	−0.3701	−0.19351
P	**<0.0001**	**0.0144**	**<0.0001**	**0.0192**	**0.0221**	**0.0039**	0.1420
FDR	**0.0014**	**0.0356**	**0.0014**	**0.0448**	**0.0489**	**0.0164**	0.2201
N	59	59	59	59	59	59	59

Coefficient of correlation with age (coeff), False Discovery Rate value (FDR) and number of evaluated DS persons are reported for total FAB and for each item. In bold significant p values are reported. * =  Spearman non-parametric correlation. ** =  Pearson parametric correlation.

**Figure 3:**
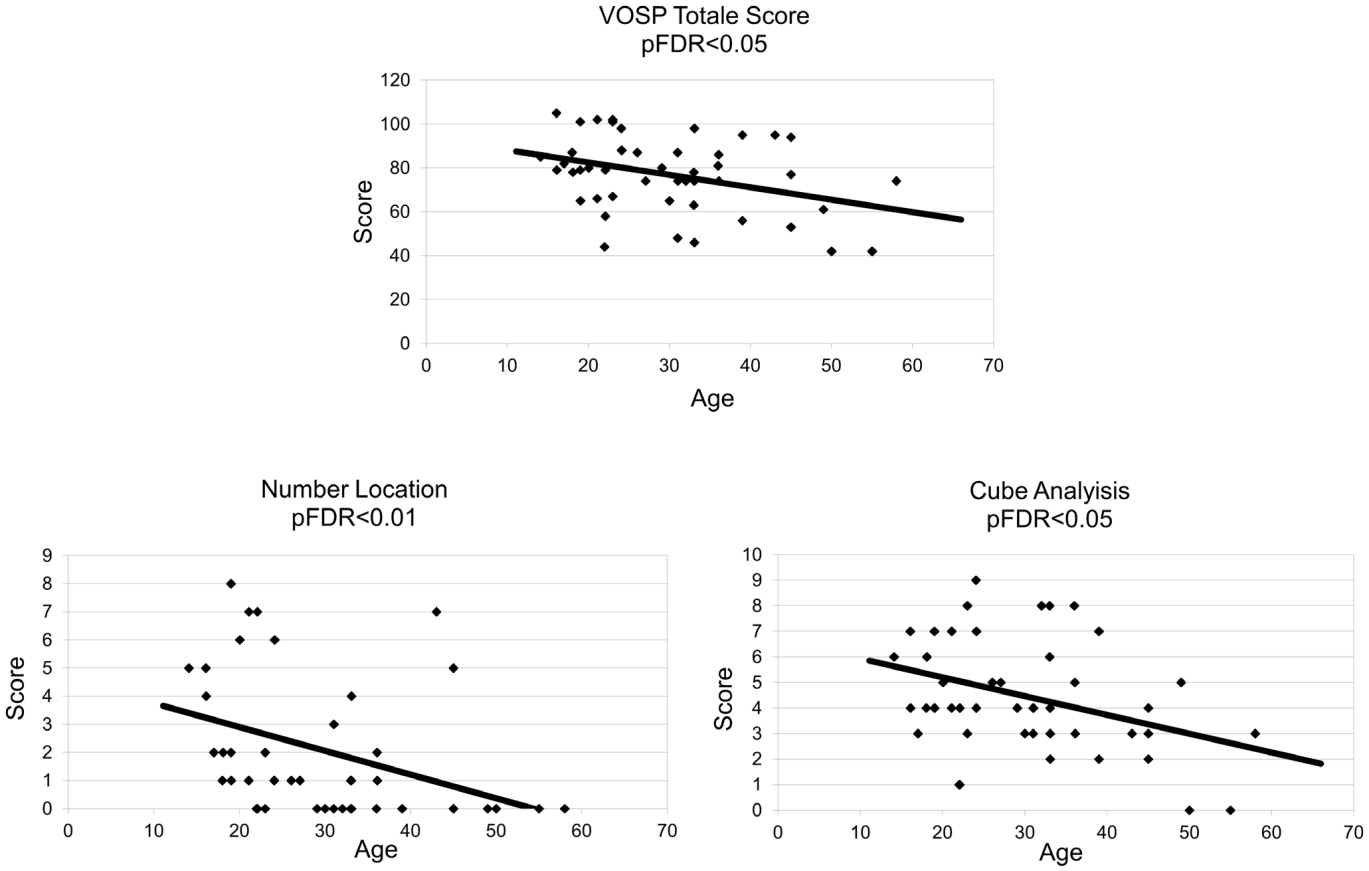
Correlations between VOSP total score, Number Location and Cube Analysis Scores, and age (expressed in years). All the values are inversely correlated with the age. pFDR = <0.05 significant, pFDR<0.01 highly significant. More details are reported in Tab le 5.

The visual-spatial skills were evaluated by using VOSP test. A total of 60 subjects underwent the test. Of these, 13 subjects did not pass the entry test (9 were over 40 years of age). We observed a significant inverse correlation with age in the score obtained for Number Location, Cube Analysis and VOSP total scores, while Silhouettes item showed only a trend (Table 5, Figure 3).

**Table 5:** Analysis of VOSP.

	Vosp total score**	Incomplete letters*	Silhouettes**	Object decision**	Progressive silhouettes*	Dot counting*	Position discrimination*	Number Location*	Cube Analysis**
Coeff	−0.36163	−0.21315	−0.30445	−0.12569	−0.10695	−0.18169	0.06748	−0.47859	−0.36158
p	**0.0125**	0.1503	**0.0375**	0.3999	0.4743	0.2216	0.6522	**0.0007**	**0.0125**
FDR	**0.0328**	0.2201	0.0750	0.4666	0.5384	0.3002	0.6848	**0.0049**	**0.0328**
N	47	47	47	47	47	47	47	47	47

Coefficient of correlation with age (coeff), False Discovery Rate value (FDR) and number of evaluated DS persons are reported for total VOSP and each item. In bold significant p values are reported. * =  Spearman non-parametric correlation. ** =  Pearson parametric correlation.

### Adaptive skills profile

**Figure 4:**
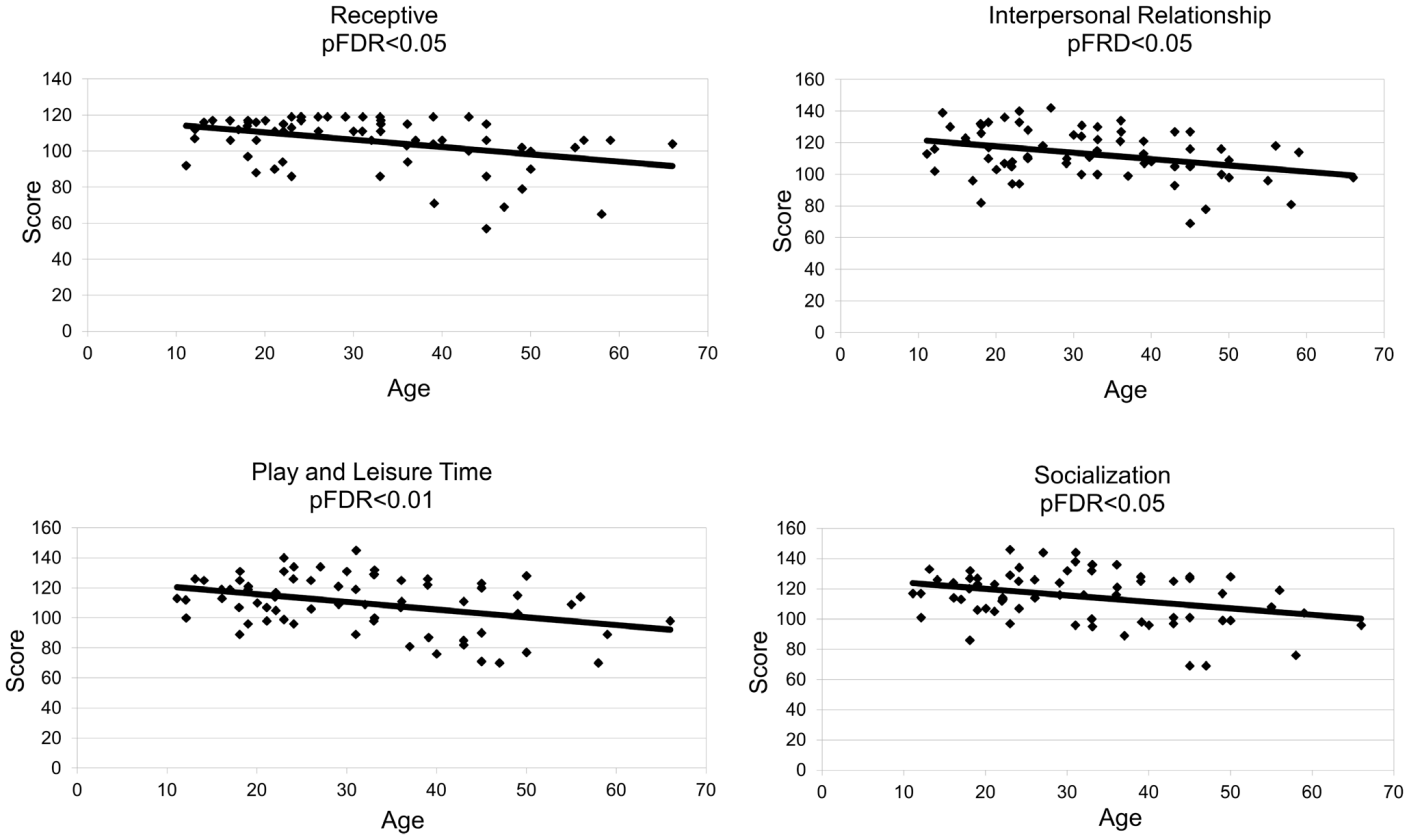
Correlations between Vineland sub-scales scores and age (expressed in years). All the values are inversely correlated with the age. pFDR = <0.05 significant, pFDR<0.01 highly significant. More details are reported in Tab 6.

The adaptive skills were assessed by using Vineland Adaptive Behaviour Scale (VABS). We observed a significant inverse correlation with age of verbal receptive language, interpersonal relationship, play/leisure time and Socialization area, while daily living skills did not change and gross motors skills showed only a trend of reduction (Table 6, Figure 4).

**Table 6:** Analysis of Vineland Adaptive Behaviour Scale (VABS).

	COMMUNICATION*	Receptive*	Expressive*	Written*	DAILY LIVING SKILLS*	Personal*	Domestic*	Community**
Coeff	−0.11820	−0.32255	−0.03727	−0.17694	−0.13596	−0.16931	−0.23711	−0.17814
P	0.3408	**0.0078**	0.7646	0.1520	0.2726	0.1708	0.0534	0.1492
FDR	0.4090	**0.0252**	0.7833	0.2201	0.3578	0.2391	0.0975	0.2201
N	67	67	67	67	67	67	67	67
	SOCIALIZATION**	Interpersonal relationship**	Play and Leisure time**	Coping Skills*	MOTOR SKILLS*	Gross motor skills*	Fine motor skills*	VINELAND TOTAL SCORE*
Coeff	−0.33064	−0.34010	−0.37675	−0.13132	−0.19005	−0.24128	−0.07252	−0.19541
P	**0.0063**	**0.0049**	**0.0017**	0.2895	0.1235	**0.0492**	0.5598	0.1130
FDR	**0.0220**	**0.0187**	**0.0089**	0.3612	0.2075	0.0939	0.6187	0.1978
N	67	67	67	67	67	67	67	67

Coefficient of correlation with age (coeff). False Discovery Rate value (FDR) and number of evaluated DS persons are reported for total VABS and each item. In bold significant p values are reported. * =  Spearman non-parametric correlation. ** =  Pearson parametric correlation

### Neuropsychiatric features

The loss of adaptive skills and dementia features were evaluated by using the DSQIID questionnaire. In the original study describing DSQIID, a score of 20 pts was reported as a cut-off for dementia [28]; we have transposed these data because of the excellent psychometric discriminant capacities shown also in the Italian version (T. Gomiero, personal communications). Five subjects scored ≥20 and they all belonged to the ≥40 years-old group (two males aged 49 and 61, three females aged 58, 63 and 70). We also divided arbitrarily the scores between 1 and 19 in two classes: 1 to 10 (considered as mild loss of skills) and 11 to 19 (considered as moderate loss of skills). The majority of 18–29 years-old DS persons (71%) were reported to have no loss of adaptive skills, compared to 53% of 30–39 years-old group and 39% of ≥40 years-old group (Table 7). At variance, no DS persons in the group 18–29 years old were found to have a score between 11 to 19, compared to the 5.9% of 30–39 years-old group and 27.8% of ≥40 years-old group. Overall, the average DSQIID total score was found to be significantly increased in the ≥40 years-old group. The most reported items, mainly beginning from the fourth decade of age, are the following: “Seems generally more tired” (12 out 58 DS persons over 18 years of age, 21%), “Appears more lazy” (11/58, 19%), “Walks slower” (10/58, 17%), “Appears generally slower” (10/58, 17%), “Loss of interest” (8/58 14%), “Slower speech” (7/58, 12%), “Appears tearful, gets more easily upset (5/58, 9%), “Social withdrawn” (4/58, 7%), “Does not initiate a conversation” (4/58, 7%), “Changed sleep pattern” (4/58, 7%), “Not confident walking” (4/58, 7%) (see DSQIID database, File S3; and Table S2 in File S1). The comparisons between 3 age groups from 18 years onward, clearly showed that neuropsychological and adaptive decay becomes more pronounced after 40 years of age, even when demented people were excluded from the analysis (Table S3 in File S1).

**Table 7:** Analysis of DSQIID score.

DSQIID score in non-demented subjects	18–29 years = C (n = 24)	30–39 years = D (n = 16)	≥40 years = E (n = 18)	P	FDR
0 (no loss of skills)*	17 (70.8%)	9 (52.9%)	7 (38.9%)		
1–10 (mild loss of skills)*	7 (29.2%)	7 (41.2%)	6 (33.3%)	**0.0410**	
11–19 (moderate loss of skills)*	0 (0.0%)	1 (5.9%)	5 (27.8%)		
Medium global score**	0.75±1.42	2.69±3.57	4.61±6.08	**0.0427** E *vs* C	0.0726

In bold significant p values are reported. * = Fisher test. ** = Non-parametric ANOVA.

## Discussion

In this study we have investigated neuropsychological, cognitive, adaptive aspects and laterality of a group of 67 persons with DS of different age, from 11 to 66 years, with no sign of dementia. Our study clearly indicates that language skills in DS persons are particularly affected in lexical and morpho-syntactic aspects, both in comprehension and in expression, and such characteristics are more pronounced in DS adults. In older DS persons, language skills resulted lower, in particular those regarding verbal comprehension (as evidenced by Token Test and Receptive subscale in VABS), capacity of denominate objects (silhouettes item of the VOSP test), semantic fluency and short-term verbal memory. In particular, short-term verbal memory assessment revealed a specific deficit of the verbal area confirming previous studies, where verbal span in DS persons was found to be lower compared to non-trisomic people with the same level of intellectual disability [38], [39].

In our sample average scores in short-term visuo-spatial memory span were found to be lower than the average score of 5 years-old children with typical development, which is 3±0,9 [40], revealing an impairment of visuo-spatial memory in DS persons. From a rehabilitation point of view, these data suggest that visual sequences must be proposed to DS persons (both in the therapeutic setting and in everyday life situations) in a repetitive and constant way, by using concrete and familiar materials, in order to facilitate learning processes through the involvement of brain associative areas. Digit forward span is found to be lower in older DS persons: in particular, a specific deficit of phonological loop, according to the working memory model of Baddeley [41], [42], might be involved.

With regard to executive functions, we observed that digit backward score in DS persons is particularly low, and in old DS persons is even lower, agreeing with the hypothesis that executive central system functions, that have a role in determining working memory functioning, are particularly affected in DS persons [39], [43]. Frontal functions show an inverse correlation with age; accordingly, we observed that the worst performance occurs in older DS persons, after 40 years of age. These data suggest a sort of anticipation of what happens in the normal population, in which a significant reduction in the FAB total score occurs mainly after the seventh decade of life, as evidenced by Appollonio et al. in an Italian adult sample [44]. The test in which there is a better score is that of Prehension (which evaluates environmental autonomy), and this is in line with what happens in the general population [44]. The sub test in which DS persons acquire the worst score is that of Verbal Fluency (thus supporting the hypothesis that language skills are more compromised than expected considering the level of cognitive impairment), whereas in adult Italian people the lower subtest score is achieved in the Similarities sub-test [44]. Interestingly, both mean scores in the various sub-tests and the mean total score of DS persons are more similar to those described by Slachevsky et al. in a group of subjects with Fronto-Temporal Dementia (FTD) compared to those observed in AD subjects [45]. This finding is of particular interest because it is believed that the symptoms of dementia in DS are related to a decay of frontal lobe functions [29], [46]. Our data suggest that this decay may be progressive over time and this becomes evident even before the overt manifestation of dementia.

In our sample left- and mixed-handed DS persons numbered 30% or even more if lower limb and eye are considered (68% and 34%, respectively), in agreement with Carlier et al. [47], who reported that DS persons are more frequently non-right-handed than the general population, where more than 90% of the persons are right-handed [48], [49]. Left-handedness can be a marker of complex dysfunction involving brain development: an increased prevalence of left-handedness has been reported in children with Autism [50], a developmental disease characterized by an abnormal brain lateralisation [51]. Our study showed no correlation between the degree of cognitive or verbal impairment and laterality (data not shown), thus suggesting that left-handedness in DS persons is probably not an additional risk factor for cognitive impairment.

In our DS group, visual perceptive functions are reduced in the older groups, as evidenced by the decrease of VOSP total score, Number location and Cube Analysis scores. This is further demonstrated by the fact that more than 50% of elderly non-demented DS persons did not pass the entry test (compared to 9% of younger people), and is not related to visual impairment. Our data showed a trend of decrease in the Silhouettes task of borderline statistical significance.

Taken together, our data suggest a decrease in right hemisphere functions, especially the Perception of spatial complex relationship in older DS persons. It is interesting to note that visual perceptive functions are impaired also in dementia. In particular, an early deficit in the Silhouettes task is reported in AD patients [52], [53].

Despite the cognitive impairment, every subject in our study had revealed a substantially good level of social adaptation (Vineland Scales) and appropriate social and cognitive skills (Social IQ, SQ). In fact, most of them achieved high SQ scores (>100) compared to persons with similar level of cognitive impairment. In agreement with the results of previous researches [54], in our sample we found a positive correlation between IQ (and other neuropsychological parameters) and SQ (data not shown), but this observation may have been influenced by recruitment bias. All DS persons involved in our study receive a great amount of care and psychological support by parental actors or caregivers, factors that are considered to have a positive influence on social adaptation and wellbeing. Vineland Scale showed a reduction in older persons of verbal receptive language, interpersonal relationship, play/leisure time and Socialization area and these data are in line with DSQIID features.

In our initial sample, a clinical picture of dementia was identified, through the questionnaire DSQIID, in 5 subjects (48, 59, 61, 63, 70 years of age). Taking into account that in our sample the subjects over 60 years of age are 4, our data are in line with what is reported in the literature: in adults with DS clinical signs and symptoms of AD were observed in 75% of people over 60 years [55], [56]. We are aware that the use of DSQIID questionnaire in persons younger than 40 years is not recommended for the diagnosis of dementia. However, we decided to use it in our investigation in younger persons with the aim of detecting early signs of loss of skills by analysing the single items one by one.

Most of the neuropsychological functions and behavioural skills measured in this study result to be lower in older subjects. As the 5 persons with overt dementia were excluded from this analysis, these results indicate that the decline of these skills starts very early and precedes by about 20–30 years the onset of dementia, which occurs mostly after the age of 60 years. Therefore, our data could suggest that possible anti-dementia treatment may not be effective in older DS persons as their neuropsychological functions and adaptive skills have already declined, while in younger DS persons, where cognitive and adaptive skills are largely maintained, it is more likely to be effective. Taken together our clinical data suggest that early cerebral deterioration involves neuronal circuits of frontal-subcortical and temporal structure, in particular dorsolateral prefrontal cortex (mental flexibility), anterior cingulate (loss of interest, lethargy) (see model proposed by Ball et al [57], medial frontal areas (word generation) [58]. At present, we can not identify with precision which is the very first function to be affected. However, DSQIID data, measuring the loss of skills over time, suggest that withdrawn from social activities/interests, fatigue, sluggishness and laziness are the more relevant symptoms detectable from 30 years onward.

The major weakness of our study is the fact that it considers a small, not randomized sample without longitudinal evaluation. Despite this limitation, we think that our data, derived from a thorough and comprehensive assessment of these persons, can offer a clear picture of the neuropsychological and adaptive state of persons with DS of different age. In fact, it seems that these data are aligned with the morphology of the brain in DS, in which a diminished proportion in the volume of the frontal and temporal lobes have been reported [59]. Age-related effects in total grey matter and in regions of the orbitofrontal and parietal cortex have also been observed [60]. Moreover, the worsening of neuropsychological functions after 40 years of age, suggested by the worse performance in neuropsychological test in the oldest group compared to the younger one, is in line with the observation that virtually all the DS persons over 40 display Alzheimer-like neuro-pathological signs [8]–[11]. The great standard deviation values found in our study can be considered as a “statistical mirror” of the great clinical variability of DS persons. In fact, despite the presence of some common features in our DS group (such as language impairment and obsessive and repetitive behaviour), the neuropsychological and behavioural profiles differ between DS persons, in terms of quantity (degree of severity) and quality (type of strengths and difficulties).

## Conclusions

In our study we found that older DS persons have lower neuropsychological functions (short memory skills, frontal lobe functions, visuo-spatial abilities) compared to younger ones, and a loss of adaptive skills. Taking into account the great clinical variability, the more apparent alarm bells detectable by care givers are mainly represented by social isolation, loss of interest and greater fatigue in daily tasks, along with a growing deficit in self-care and in the ability to understand social rules in everyday life. We underline that a shared basis of these features is the impairment in language skills, which is particularly pervasive, appears to be specific and seems to be unrelated to the cognitive profile of the person; it constitutes a key element to be considered in this framework (especially in the component of understanding). We suggest that a) neurological, psychiatric and neuropsychological evaluations of DS persons, performed on a regular basis, may be a very useful tool in order to detect the early stages of the decline in neurocognitive functions; b) in DS adults, a combined therapy including neuro-cognitive rehabilitation and psycho-pharmacological interventions might be very useful in order to prevent or delay the onset of dementia, provided it is started earlier than the age of onset of overt cognitive decline, *e.g.* as soon as the fourth decade of age (or even earlier).

## Supporting Information

File S1 **Supporting files.** Cognitive, neuropsychological and adaptive assessment. Details and references on the tests used for these assessments. Table S1, DS persons who scored “0” at each neuropsychological test. Table S2, Analysis of DSQIID data indicating a worsened condition per single item. Numbers of persons are reported per age group and in the total sample. Worsening is considered when the answer to the item is “always but worse”, or “new symptoms”, or “yes”. Table S3, A: Neuropsychological and cognitive test; B Vineland Adaptive Behaviour Scale. Comparison between the means values in 3 age groups over 18 years, performed using the ANOVA or the equivalent non parametric test (* = Kruskal-Wallis). The values are expressed as means ± SD. False Discovery Rate values (FDR) are reported for each item. In bold significant p values are reported. In square brackets the number of subjects evaluated for groups subdivided for age. 18–29 years group =  C; 30–39 years group =  D; ≥40 years group = E.
(DOCX)

File S2 **General DS Database in excel format.**
(XLSX)

File S3 **DS Database for the DSQIID data in excel format.**
(XLSX)
