# Supplementary material for: Age-Related Changes of Adaptive and Neuropsychological Features in Persons with Down Syndrome
Source: PLoS One. 2014 Nov 24;9(11):e113111. doi: 10.1371/journal.pone.0113111 (PMC4242614; doi:10.1371/journal.pone.0113111)
Supplement: File S1 — Supporting files. Cognitive, neuropsychological and adaptive assessment. Details and references on the tests used for these assessments. Table S1, DS persons who scored “0” at each neuropsychological test. Table S2, Analysis of DSQIID data indicating a worsened condition per single item. Numbers of persons are reported per age group and in the total sample. Worsening is considered when the answer to the item is “always but worse”, or “new symptoms”, or “yes”. Table S3, A: Neuropsychological and cognitive test; B Vineland Adaptive Behaviour Scale. Comparison between the means values in 3 age groups over 18 years, performed using the ANOVA or the equivalent non parametric test (* = Kruskal-Wallis). The values are expressed as means ± SD. False Discovery Rate values (FDR) are reported for each item. In bold significant p values are reported. In square brackets the number of subjects evaluated for groups subdivided for age. 18–29 years group = C; 30–39 years group = D; ≥40 years group = E. (DOCX) [file pone.0113111.s001.docx]

**Ghezzo et al. Supplemental material:**

**Cognitive, neuropsychological and adaptive assessment**

1. WISC-III: Wechsler Intelligence Scale revised for children between the ages of 6 years and 16 years and 11 months [1].
2. WAIS-R: Wechsler Adult Intelligence Scale - Revised for subjects aged 17 years or more [2].
3. Short-term Verbal Memory: Digit Span subtests (WAIS-R) were administered. Digit Span subtest is composed by two different tasks: Digit Forward (repetition of a series of numbers in their original order) and Digit Backward (repetition of a series of numbers in reverse).
4. Spatial Span (Corsi’s Block Test - [3]: the test evaluates the visuo-spatial short-term memory. Visuo-spatial span refers to the longest series of items that the subject can remember in the same order immediately after presentation.
5. Categorical fluency [4] (modified): Semantic fluency test of lexical recovery. Each subject nominates in one minute of time the largest number of elements belonging to 4 categories (colours, animals, fruits, cities).
6. Token Test [5]: test of the syntactic and propositional comprehension in receptive language. The score ranges for 0 to 36 and resulted from the sum of correct answers of the subject.
7. Phonological fluency [6] (modified): test of lexical recovery based on phonological clue (words start with sound /a/; /s/; /f/). Subject have one minute for each phonological category to name the largest number of words.
8. Tower of London: The Tower of London (TOL) is a neuropsychological test originally developed by [7] and used to assess problem-solving and planning skills. It is based on the use of a tool consisting of three pegs of different lengths, in which are strung three balls (one red, one green and one blue): the subject must move these marbles in a number of moves in order to get the configuration indicated by the examiner. There are 12 problems of different difficulties: we chose to collect only the “perfect solution score” (the number of problems correctly solved in the first trial, using the minimum number of moves) because this parameter has been related to planning ability and to the formulation of strategies [8].
9. Frontal Assessment Battery (FAB) [9]: this test is a short neuropsychological tool devised for the fast bedside screening of frontal functions. The FAB consists of six subtests, each exploring functions related to the frontal lobes: conceptualization (similarities task), mental flexibility (phonological fluency task), motor programming (Luria’s motor series), sensitivity to interference (conflicting instructions task), inhibitory control (go-no-go task), and environmental autonomy (prehension behaviour). An Italian version of the FAB [10] was administered to our study group by CC.
10. The Visual Object and Space Perception Battery – VOSP [11]: the test consists of 4 subtests that measure visual object perception (Incomplete Letters; Silhouettes; Object Decision; Progressive Silhouettes) and 4 visual space perception tasks (Dot Counting; Position Discrimination; Number Location; Cube Analysis). The VOSP battery is preceded by a basic visual screening task that checks whether the subject’s visual sensory capacities are sufficiently intact to permit further examination (Shape Detection Screening Test- SS.VO.). VOSP was administered by CC.
11. Laterality assessment: was performed by an experienced psychomotricist (CC). It was carried out a qualitative assessment of the prevalence of side (laterality) through objective evidence such as: Upper Limb: Pen (use), hammer (use), Comb (use), unscrew / screw, grasp his wrist, his fists Overlap, Overlap indices. Lower Limb: Kicking a ball, climb on a bench, jumping / balancing on one foot, Stand in an upright position. Eye: look through a telescopic tool and through a hole; to wink. The laterality of the subjects was defined as follows: left when all the items were performed with the left limb or eye; right when all the items were performed with the right limb or eye. Mixed laterality was defined when there was a change in the limb/eye while performing a task, or when the tasks were not performed with the same limb/eye.
12. Vineland Adaptive Behaviour Scales (VABS) [12]: the test assesses personal autonomy and social responsibility of individuals from birth to adulthood through questions designed to assess the skills actually possessed by the subject at the time of evaluation. The scales are used with both non-disabled and disabled persons. The questionnaire in its complete form consists of 540 items that include the following areas: communication, daily living skills, socialization, motor skills. The questionnaire was directly administered to parents, or whoever takes care of the DS persons, by MCS, AG and AP.
13. DSQIID Questionnaire (Dementia Screening Questionnaire for Individuals with Intellectual Disabilities, [13] to identify predictors of dementia, which involves assigning a score for each question: a total score equal to or greater than 20 is indicative of dementia. DSQIID has been evaluated in adults with DS and had excellent inter-rater reliability, sensitivity, and specificity [14,15] and an excellent psychometric characteristics showed also in the Italian version (T. Gomiero, personal communication).

**References**

1. Wechsler D., WISC-III: Wechsler intelligence scale for children III, New York, The Psychological Corporation, 1991 –Adattamento italiano di Arturo Orsini e Laura Picone O.S. Organizzazioni Speciali, Firenze 2006
2. Wechsler, D. (1981), WAIS-R. Scala d’Intelligenza Wechsler per Adulti-Riveduta. Tr. it. O.S. Organizzazioni Speciali, Firenze 1997
3. Milner B (1971) Interhemispheric differences in the localization of psychological processes in man. Br Med Bull 27: 272-277.
4. Orsini A, Grossi D, Capitani E, Laiacona M, Papagno C, Vallar G (1987) Verbal and Spatial Immediate Memory Span. Italian Journal of Neurological Science 8: 539-548.
5. De Renzi E, Vignolo LA (1962) The token test: a sensitive test receptive disturbances in aphasics. Brain 85: 665-78.
6. Novelli G, Papagno C, Capitani E, Laiacona N, Vallar G, Cappa S.F (1986) Tre test clinici di ricerca e produzione lessicale. Taratura su soggetti normali. Archivio di Psicologia, Neurologia e Psichiatria 47: 477-506.
7. Shallice T (1982) Specific impairments of planning. Philosophical Transactions, Royal Society of London. B. Biological Science 298: 199–209.
8. Levin H, Culhane K ,Hartmann H, Evankovich K, Mattson A J, Harward H et al. (1991) Developmental changes in performance on tests of purported frontal lobe functioning. Developmental Neuropsychology 7: 377–395
9. Dubois B, Slachevsky A, Litvan I, Pillon B (2000) The FAB: a frontal assessment battery at bedside. Neurology 55: 1621-1626
10. Appollonio I, Piamarta F, Isella V, Leone M, Consoli T, Nichelli P (2004) Strumenti di lavoro: la Frontal Assessment Battery (FAB). Demenze 7: 28–33
11. Warrington EK, Merle J (1991) The Visual Object and Space Pereption Battery, Thames Valley Test Company.
12. Sparrow SS, Balla DA, Chicchetti DV (2003) Vineland adaptive behavior Scales: intervista- forma completa. Adattamento italiano a cura di Giulia Balboni e Luigi Pedrabissi. O.S Organizzazioni Speciali; Firenze
13. Deb S, Hare M, Prior L, Bhaumik S (2007a) Dementia screening questionnaire for individuals with intellectual disabilities. British Journal of Psychiatry 190: 440-444.
14. Deb S, Hare M, Prior L (2007b) Symptoms of dementia among adults with Down’s syndrome: a qualitative study. Journal of Intellectual Disability Research 51: 726–739.
15. Parmenter T R (2008) The dementia screening questionnaire for individuals with intellectual disabilities has high sensitivity and specificity in adults with Down's syndrome. Evidence Based Mental Health 11: 11.

**Table S1**: DS persons who scored “0” at each neuropsychological test

| **Test** | **Number of DS persons**  **who scored 0 (null)** | **Number of DS persons**  **who performed the test** | **%** |
| --- | --- | --- | --- |
| **Tower of London** | 15 | 62 | 24% |
| **Digit span forward** | 9 | 65 | 13% |
| **Digit span backward** | 36 | 65 | 55% |
| **Spatial span (Corsi)** | 13 | 63 | 21% |
| **Token test (comprehension)** | 4 | 65 | 6% |
| **Phonemic fluency** | 13 | 65 | 20% |
| **Semantic fluency** | 5 | 65 | 8% |
| **Frontal Assessment Battery FAB total score** | 1 | 59 | 2% |
| **FAB similarity** | 21 | 59 | 36% |
| **FAB verbal fluency** | 35 | 59 | 60% |
| **FAB motor series** | 8 | 59 | 14% |
| **FAB conflictual instructions** | 28 | 59 | 48% |
| **FAB go-no go** | 30 | 59 | 51% |
| **FAB prehension** | 5 | 59 | 8% |
| **VOSP Entry test S.S.-V.O. (persons who did not pass the entry test)** | 13 | 60 | 22% |
| **VOSP subtest 5 dot counting** | 4 | 47 | 9% |
| **VOSP subtest 6 position discrimination** | 2 | 47 | 4% |
| **VOSP subtest 7 number location** | 18 | 47 | 38% |
| **VOSP subtest8 cube analysis** | 2 | 47 | 4% |

**Table S2**: Analysis of DSQIID data indicating a worsened condition per single item. Numbers of persons are reported per age group and in the total sample. Worsening is considered when the answer to the item is “always but worse”, or “new symptoms”, or “yes”.

| ITEM | 18-29 years | 30-39 years | ≥40 years | total |
| --- | --- | --- | --- | --- |
|  |  |  |  |  |
| Cannot wash and/or bathe without help | 0 | 0 | 3 | 3 |
| Cannot dress without help | 0 | 0 | 1 | 1 |
| Dresses inappropriately | 0 | 0 | 0 | 0 |
| Undress inappropriately | 0 | 0 | 0 | 0 |
| Needs help eating | 0 | 0 | 0 | 0 |
| Needs help using the bathroom | 0 | 0 | 2 | 2 |
| Incontinent | 1 | 0 | 3 | 4 |
| Does not initiate conversation | 1 | 2 | 1 | 4 |
| Cannot find words | 0 | 1 | 1 | 2 |
| Cannot follow simple instruction | 0 | 0 | 1 | 1 |
| Cannot follow more than one instruction at a time | 0 | 1 | 1 | 2 |
| Stops in the middle of a task | 0 | 0 | 2 | 2 |
| Cannot read | 0 | 0 | 0 | 0 |
| Cannot write | 1 | 0 | 1 | 2 |
| Changed sleep pattern | 1 | 2 | 1 | 4 |
| Wakes frequently at night | 0 | 1 | 0 | 1 |
| Confused at night | 0 | 0 | 1 | 1 |
| Sleeps during the day | 0 | 0 | 0 | 0 |
| Wanders at night | 0 | 0 | 1 | 1 |
| Cannot find way in familiar surroundings | 0 | 0 | 2 | 2 |
| Wanders | 0 | 0 | 1 | 1 |
| Loses track of time | 0 | 0 | 3 | 3 |
| Not confident walking over small tracks, line on the grounds, or uneven surfaces | 0 | 1 | 3 | 4 |
| Unsteady walk, loses balance | 0 | 1 | 1 | 2 |
| Cannot walk unaided | 0 | 0 | 0 | 0 |
| Cannot recognise familiar persons | 0 | 0 | 0 | 0 |
| Cannot remember names of familiar persons | 0 | 0 | 0 | 0 |
| Cannot remember recent events | 0 | 0 | 0 | 0 |
| Withdraws from social activities | 0 | 2 | 2 | 4 |
| Withdraws from person | 0 | 0 | 0 | 0 |
| Loss of interest in hobbies and activities | 2 | 2 | 4 | 8 |
| Seems to go into own world | 0 | 0 | 3 | 3 |
| Obsessive or repetitive | 1 | 0 | 1 | 2 |
| Hides or hoards objects | 1 | 0 | 2 | 3 |
| Loses objects | 0 | 0 | 2 | 2 |
| Puts familiar things in wrong places | 0 | 0 | 0 | 0 |
| Does not know what to do with familiar objects | 0 | 0 | 0 | 0 |
| Appears insecure | 0 | 1 | 0 | 1 |
| Appears anxious | 1 | 1 | 0 | 2 |
| Appears depressed | 1 | 2 | 0 | 3 |
| Shows aggression | 0 | 0 | 0 | 0 |
| Fits/epilepsy | 0 | 0 | 1 | 1 |
| Talks to self | 0 | 0 | 2 | 2 |
| Lost some skills (e.g. brushing teeth) | 0 | 0 | 4 | 4 |
| Speaks (or signs) less | 1 | 3 | 3 | 7 |
| Seems generally more tired | 1 | 5 | 6 | 12 |
| Appears tearful, gets more easily upset | 1 | 0 | 4 | 5 |
| Appears generally slower | 3 | 4 | 3 | 10 |
| Slower speech | 1 | 2 | 4 | 7 |
| Appears more lazy | 2 | 5 | 4 | 11 |
| Walks slower | 1 | 5 | 4 | 10 |
| Generally appears more forgetful | 0 | 0 | 3 | 3 |
| Generally appears more confused | 0 | 0 | 4 | 4 |

**Table S3**. A: Neuropsychological and cognitive test; B Vineland Adaptive Behaviour Scale.

Comparison between the means values in 3 age groups over 18 years, performed using the ANOVA or the equivalent non parametric test (*=Kruskal-Wallis). The values are expressed as means ± SD. False Discovery Rate values (FDR) are reported for each item. In bold significant p values are reported. In square brackets the number of subjects evaluated for groups subdivided for age. 18-29 years group = C; 30-39 years group = D; ≥40 years group= E.

**A**

|  | **18-29 years= C** | **30-39 years= D** | **≥40 years= E** | **P=** | **FDR** | **(p<0,05)** |
| --- | --- | --- | --- | --- | --- | --- |
| **SHORT MEMORY SPAN** |  |  |  |  |  |  |
| **Spatial span (Corsi Test) * [C=24, D=14, E=17]** | 2,75 ± 1,39 | 2,14 ± 1,46 | 1,47 ± 1,18 | **0,0134** | **0,0387** | E vs C |
| **Digit span forward * [C=24, D=15, E=18]** | 2,46 ± 1,22 | 2,40 ± 1,30 | 2,00 ± 1,19 | 0,4154 | 0,4903 |  |
|  |  |  |  |  |  |  |
| **LANGUAGE** |  |  |  |  |  |  |
| **Semantic fluency [C=24, D=15, E=18]** | 29,79 ± 13,21 | 25,50 ± 12,74 | 15,76 ± 10,91 | **0,0032** | **0,0203** | E vs C |
| **Comprehension (Token test)*** | 21,35 ± 8,52 | 19,50 ± 9,05 | 11,92 ± 6,87 | **0,0012** | **0,0104** | E vs D; E vs C |
|  |  |  |  |  |  |  |
| **EXECUTIVE FUNCTIONS** |  |  |  |  |  |  |
| **Phonemic fluency* [C=24, D=16, E=17]** | 7,79 ± 6,65 | 6,19 ± 4,58 | 1,53 ± 2,00 | **0,0005** | **0,0091** | E vs D; E vs C |
| **Digit span backward* [C=24, D=15, E=18]** | 1,17 ± 1,37 | 1,47 ± 0,92 | 0,39 ± 0,92 | **0,0201** | 0,0523 |  |
| **Tower of London (n° perfect answers)***  **[C=22, D=16, E=17]** | 4,23 ± 2,65 | 3,31 ± 3,59 | 1,35 ± 2,29 | **0,0110** | **0,0358** | E vs C |
|  |  |  |  |  |  |  |
| **FRONTAL ASSESSMENT BATTERY-FAB**  **[C=19, D=15, E=17]** |  |  |  |  |  |  |
| **FAB total score** | 8,37 ± 3,71 | 8,80 ± 3,43 | 4,59 ± 2,50 | **0,0007** | **0,0091** | E vs D; E vs C |
| **Similarity*** | 1,10 ± 1,00 | 1,07 ± 0,88 | 0,65 ± 0,86 | 0,2683 | 0,3488 |  |
| **Phonological fluency*** | 0,68 ± 0,75 | 0,53 ± 0,64 | 0,06 ± 0,24 | **0,0078** | **0,0338** | E vs D; E vs C |
| **Motor series*** | 1,74 ± 0,93 | 2,27 ± 1,03 | 1,23 ± 1,25 | **0,0360** | 0,0728 |  |
| **Conflictual instructions*** | 1,05 ± 1,08 | 0,87 ± 1,06 | 0,35 ± 0,61 | 0,0863 | 0,1402 |  |
| **Go-no go*** | 1,10 ± 1,00 | 1,07 ± 1,10 | 0,18 ± 0,39 | **0,0039** | **0,0203** | E vs D; E vs C |
| **Prehension behaviour*** | 2,68 ± 0,75 | 3,00 ± 0,00 | 2,12 ± 1,32 | **0,0364** | 0,0728 |  |
|  |  |  |  |  |  |  |
| **VISUAL OBJECTS and SPACE PERCEPTIONS BATTERY-VOSP (cut-off) [C=20, D=15, E=8]** |  |  |  |  |  |  |
| **Incomplete Letters (17)*** | 16,40 ± 2,44 | 16,40 ± 2,26 | 14,13 ± 4,64 | 0,4765 | 0,5387 |  |
| **Silhouettes (16)** | 13,80 ± 4,53 | 9,07 ± 3,88 | 11,00 ± 4,66 | **0,0098** | **0,0358** | D vs C |
| **Object Decision (15)** | 12,30 ± 3,25 | 12,60 ± 2,75 | 11,50 ± 3,74 | 0,7306 | 0,7598 |  |
| **Progressive Silhouettes (14)*** | 9,25 ± 3,64 | 8,33 ± 4,05 | 9,25 ± 2,31 | 0,7203 | 0,7598 |  |
| **Dot Counting (8 )*** | 7,70 ± 2,58 | 7,67 ± 2,69 | 4,50 ± 3,74 | 0,0739 | 0,1281 |  |
| **Position Discrimination (18)*** | 14,05 ± 3,20 | 13,53 ± 4,52 | 12,88 ± 6,51 | 0,9887 | 0,9887 |  |
| **Number Location (7)*** | 2,70 ± 2,77 | 1,00 ± 1,36 | 1,50 ± 2,83 | 0,0510 | 0,0947 |  |
| **Cube Analysis (6)** | 5,00 ± 2,18 | 4,67 ± 2,19 | 2,50 ± 1,77 | **0,0249** | 0,0589 |  |
| **Total score** | 81,20 ± 15,87 | 73,27 ± 15,52 | 67,25 ± 21,19 | 0,1199 | 0,1732 |  |
|  |  |  |  |  |  |  |
| **WECHSLER SCALE** |  |  |  |  |  |  |
| **VERBAL IQ* [C=21, D=10, E=5]** | 53,43 ± 13,02 | 51,60 ± 12,91 | 33,60 ± 20,02 | 0,0991 | 0,1516 |  |
| **PERFORMANCES IQ*[C=21, D=10, E=5]** | 51,38 ± 12,49 | 52,90 ± 12,44 | 36,20 ± 23,72 | 0,4020 | 0,4909 |  |
| **TOTAL IQ* [C=21, D=10, E=5]** | 49,71 ± 12,69 | 48,80 ± 11,84 | 33,20 ± 19,60 | 0,2430 | 0,3325 |  |

**B**

| **VINELAND ADAPTIVE BEHAVIOUR SCALE [C=24, D=17, E=18]** | **18-29 years= C** | **30-39 years= D** | **≥40 years= E** | **P=** | **FDR** | **(p<0,05)** |
| --- | --- | --- | --- | --- | --- | --- |
| **COMMUNICATION*** | 109,50 ± 14,18 | 107,71 ± 16,10 | 100,61 ± 16,51 | 0,1395 | 0,1694 |  |
| **Receptive*** | 110,50 ± 10,90 | 106,94 ± 12,89 | 96,17 ± 18,19 | **0,0071** | **0,04** | E vs D; E vs C |
| **Expressive*** | 107,04 ± 14,23 | 104,71 ± 14,90 | 101,78 ± 15,47 | 0,4269 | 0,4536 |  |
| **Written*** | 111,25 ± 14,90 | 110,41 ± 15,32 | 99,44 ± 15,49 | 0,0672 | 0,1039 |  |
| **DAILY LIVING SKILLS*** | 118,29 ± 10,23 | 117,71 ± 17,11 | 106,78 ± 16,91 | **0,0417** | 0,0726 | E vs D; E vs C |
| **Personal*** | 110,96 ± 8,57 | 110,47 ± 11,90 | 101,44 ± 15,15 | 0,0965 | 0,1367 |  |
| **Domestic*** | 121,46 ± 10,51 | 116,35 ± 17,94 | 107,56 ± 15,45 | **0,0183** | 0,0622 | D vs C |
| **Community** | 116,63 ± 18,10 | 120,53 ± 20,87 | 104,22 ± 18,03 | **0,0324** | 0,0726 |  |
| **SOCIALIZATION** | 119,04 ± 13,97 | 119,88 ± 17,90 | 103,28 ± 18,79 | **0,0050** | **0,0402** | E vs D; E vs C |
| **Interpersonal relationship** | 116,46 ± 15,79 | 116,47 ± 11,90 | 103,22 ± 16,05 | **0,0104** | **0,0442** | E vs D; E vs C |
| **Play and Leisure time** | 115,00 ± 14,12 | 114,12 ± 18,30 | 96,17 ± 19,70 | **0,0016** | **0,0272** | E vs D; E vs C |
| **Coping Skills*** | 118,83 ± 12,52 | 119,18 ± 16,57 | 110,22 ± 17,35 | 0,1858 | 0,2106 |  |
| **MOTOR SKILLS*** | 110,67 ± 6,59 | 109,00 ± 11,65 | 104,72 ± 11,03 | 0,1243 | 0,16 |  |
| **Gross Motor Skills*** | 109,83 ± 6,91 | 107,59 ± 11,68 | 103,11 ± 9,46 | **0,0313** | 0,0726 | E vs D; E vs C |
| **Fine Motor Skills*** | 110,75 ± 7,93 | 108,76 ± 12,14 | 105,44 ± 13,15 | 0,5222 | 0,5222 |  |
| **Vineland Total Score (SQ)*** | 116,75 ± 10,81 | 116,00 ± 15,01 | 105,22 ± 16,15 | **0,0407** | 0,0726 |  |
